# Re-Evaluation of ELISA for the Detection of Bovine Tuberculosis and a New Proposal for Its Use in Eradication Efforts on Outbreak Farms

**DOI:** 10.3390/pathogens14040331

**Published:** 2025-03-30

**Authors:** Chan-Ho Park, Jaemung Kim, Yun-Ho Jang, Sehyun Son, Sungweon Ryoo, Jung-Ho Kim, Sang-Min Won, Kyu-Wook Kim, Sungwon Hong, Bo-Young Jeon, Son-Il Pak, Byung-Il Yoon

**Affiliations:** 1 College of Veterinary Medicine and Institute of Veterinary Science, Kangwon National University, Chuncheon 24341, Republic of Korea; 2 Gangwon State Veterinary Service & Research Institute, Chuncheon 24203, Republic of Korea; 3 Bacterial Disease Division, Department of Animal & Plant Health Research, Animal and Plant Quarantine Agency, Gimcheon 39660, Republic of Korea; kimjm88@korea.kr (J.K.); cllaric@naver.com (Y.-H.J.); scriptbar@korea.kr (S.S.); 4 Clinical Research Center, Masan National Tuberculosis Hospital, Changwon 51755, Republic of Korea; viweon@gmail.com; 5 Bio-Contents Operation Division, BIONOTE, Inc., Hwaseong-si 18449, Republic of Korea; jungho@bionote.co.kr; 6 Agriculture & Livestock Division, Ulsan Metropolitan City, Ulsan 44675, Republic of Korea; wonsangmin@korea.kr; 7 Solvet, Inc., Anseong-si 17609, Republic of Korea; ironman@solvet.co.kr; 8 KogeneBiotech Co., Geumcheon-gu, Seoul 08507, Republic of Korea; swhong@kogene.co.kr; 9 Department of Biomedical Laboratory Science, College of Software and Digital Healthcare Convergence Yonsei University, Wonju 26493, Republic of Korea; bojeon87@gmail.com

**Keywords:** bovine tuberculosis, South Korea, ELISA, seven days post-PPD

## Abstract

Bovine tuberculosis (bTB) is a zoonotic infectious disease and a chronic wasting illness. Accordingly, detecting and eradicating bTB remains a significant challenge in South Korea. This study evaluated the efficacy of a modified enzyme-linked immunosorbent assay (ELISA) protocol for detecting bTB in cattle. The protocol included two ELISA tests: one performed on the day of purified protein derivative (PPD) inoculation and another seven days post-inoculation. Results show a significant increase in ELISA detection rates, from 11% to 76%, particularly in cattle that tested positive for the tuberculin skin test (TST) and/or interferon-gamma (IFN-γ) assays (*p* < 0.0001). Notably, some cattle that were negative or had doubtful results in TST and IFN-γ assays transitioned to ELISA positive post-PPD inoculation. Additionally, some cattle identified as positive only by ELISA (S/*p* value ≥ 0.3) were confirmed to have bTB through gross examination or real-time reverse transcription polymerase chain reaction (rRT-PCR). The proposed protocol was validated in bTB outbreak farms using S/*p* thresholds of 0.3 (PPD inoculation day) and 0.5 (seven days post-PPD), enabling the detection of infected cattle missed by TST and IFN-γ assays. Implementing this approach successfully eradicated bTB in outbreak farms with minimal culling. These findings highlight the potential of incorporating sequential ELISA tests to enhance bTB detection and support eradication efforts.

## 1. Introduction

Bovine tuberculosis (bTB) is a zoonotic infectious disease caused by *Mycobacterium bovis*. Severe clinical symptoms of bTB include fever, weakness, weight loss, enlarged lymph nodes, anemia, and reduced milk yield. However, most bTB cases remain asymptomatic, necessitating diagnostic testing for confirmation. Farms experiencing bTB outbreaks have struggled with this disease for several decades. The most widely used diagnostic test for bTB detection in farms has been the tuberculin skin test (TST). In 2013, countries with advanced livestock management systems, such as the United Kingdom and Australia, introduced the interferon-gamma (IFN-γ) assay due to its high sensitivity and specificity. Both TST and IFN-γ assays detect bTB based on cellular immune responses, making them effective in identifying infections in early and middle stages [1,2]. However, they are not very useful in detecting late stages of bTB infection and may display a false negative due to anergy as a result of the other infection being induced by an immunosuppressive conditions or drugs [3]. Since 2013, South Korea has implemented the IFN-γ assay alongside the TST to enhance the efficiency of bTB testing. An epidemiological study conducted by the Korean Animal and Plant Quarantine Agency (QIA) analyzed 1260 farms affected by tuberculosis outbreaks between 2012 and 2017. The study revealed that 24.4% (308/1260) of these farms experienced three or more tuberculosis outbreaks, and 7.6% (96/1260) of farms previously declared tuberculosis-free faced reinfection. Furthermore, cattle over 36 months old accounted for 49.1% of the total bTB-positive cases. These older cattle often display chronic disease symptoms, contributing to recurrent farm outbreaks [4]. As outlined above, bTB is a chronic disease, and despite detecting infected animals, outbreaks can recur years later due to latent, false-negative carriers. A cellular immunity test is insufficient to detect latent infections in cattle, thereby limiting eradication efforts. The enzyme-linked immunosorbent assay (ELISA) presents a promising alternative, as it is based on humoral immune response detection, making it particularly effective for late-stage bTB infections. Additionally, ELISA can detect anergic cattle suffering from immunosuppressive conditions, where TST and IFN-γ assays may fail [5,6,7]. While ELISA was temporarily implemented between 2009 and 2013, it was eventually replaced by IFN-γ assays due to low agreement with TST and poor detection rates [8,9]. Per the World Organisation for Animal Health (WOAH)’s Manual of Diagnostic Tests and Vaccines for Terrestrial Animals, ELISA is particularly useful in diagnosing anergic T cells, and ELISA methods utilizing tuberculosis-specific antigens (e.g., MPB70 and MPB83) have demonstrated high specificity [10,11,12,13].

Furthermore, previous studies have reported a significant increase in bTB detection rates when ELISA was performed post-TST in bTB-positive cattle [14,15,16,17]. Given the challenges associated with current bTB diagnostic methods [18,19], this study aims to comprehensively evaluate the role of ELISA in bTB detection and eradication. Specifically, we seek to investigate the underlying reasons for ELISA’s initially lower detection rates in comparison to TST and IFN-γ assays, as well as to assess its diagnostic agreement with these conventional tests. Furthermore, we aim to examine changes in ELISA results before and after purified protein derivative (PPD) inoculation in bTB-positive cattle in order to determine whether this approach enhances detection sensitivity.

Based on these findings, we propose a novel ELISA-based diagnostic protocol that can improve bTB eradication efforts in outbreak farms, facilitating more effective disease control with minimal culling.

## 2. Materials and Methods

### 2.1. Diagnostic Procedures Section

#### 2.1.1. Tuberculin Skin Test (TST)

According to the product manual, the PPD solution (a purified protein derivative antigen, BoviShot^®^, CAVAC, Daejeon, Republic of Korea) for TST was intradermally injected into the tail of the cattle. The skin-fold thickness at the inoculation site was measured between 48 and 72 h after PPD inoculation. The difference in skin-fold thickness was interpreted as follows: The difference in skin-fold thickness was considered positive when it was 5 mm or more, doubtful if it was between 3 mm and 5 mm, and negative when it was less than 3 mm.

#### 2.1.2. Interferon-Gamma Assay (IFN-γ)

The whole blood was collected into heparinized tubes and stored at room temperature, with cultivation initiated within 12 h. It was aliquoted into a 24-well cell culture plate, and PBS, PPD-A, and PPD-B antigen stimulants were added to each well. The plate was then incubated at 37 °C for 16–24 h.

After incubation, the samples were centrifuged, and plasma was collected by separating the supernatant. Following the manufacturer’s protocol (TB-Feron^®^, BIONOTE, Hwaseong-si, Republic of Korea), the plasma was mixed with sample diluent and aliquoted into an antibody adsorption plate. Positive and negative controls were included, and the plate was incubated at 37 °C. Next, amplification and conjugate solutions were added, followed by washing and substrate addition. Finally, 100 µL of stop solution was added, and absorbance was measured at 450 nm. The results were interpreted based on the response to the stimulating antigens: A positive result was determined when the difference in absorbance between the PPD-B-stimulated plasma and the negative control plasma was ≥0.1 and if the difference between PPD-B and PPD-A was also ≥0.1. A negative result was indicated if the absorbance differences were below these thresholds.

#### 2.1.3. ELISA

The BTB Ab ELISA 2.0 kit (BTB Ab ELISA 2.0^®^, BIONOTE, Hwaseong-si, Republic of Korea) was used to detect bovine tuberculosis (bTB) antibodies. The test was performed following the product manual. First, 50 µL of test samples (bovine serum), positive controls, and negative controls were added to each well. Then, 50 µL of enzyme conjugate was added to each well and incubated at 37 °C for one hour. Each well was washed six times with 350 µL of diluted washing solution. After washing, 100 µL of substrate solution was added to each well and incubated at room temperature (15–25 °C) for 15 min in the dark. Finally, 100 µL of stop solution was added, and absorbance was measured using a spectrophotometer at 450 nm with a reference wavelength of 620 nm.

The ELISA antibody results, expressed as the sample/positive ratio (S/*p* value), were interpreted as follows: values below 0.3 were considered negative, values between 0.3 and 0.5 were considered doubtful, and values above 0.5 were considered positive.

#### 2.1.4. Tissue Sample Collection and Real-Time Reverse Transcription Polymerase Chain Reaction (rRT-PCR)

Tissue samples suspected of *Mycobacterium bovis* infection, including pharyngeal and pulmonary mediastinal lymph nodes, were collected postmortem. Five to ten grams of each tissue sample were placed into sterile stainless steel containers and cut into small pieces using sterile scissors. The tissue was then homogenized in 10 mL of phosphate-buffered saline (PBS) using a homogenizer. The homogenized tissue was transferred to centrifuge tubes, and an equal volume of 10% oxalic acid was added for decontamination. The mixture was incubated at room temperature for 10 min. Following incubation, the samples were filtered sequentially using 70 µm and 40 µm cell strainers. The filtered samples were centrifuged at 3000× *g* for 10 min. The supernatant was discarded, and the pellet was resuspended in 2 mL of PBS and vortexed vigorously.

DNA was extracted using the Viral Gene-spin™ Viral DNA/RNA Extraction Kit (iNtron, Seongnam-si, Republic of Korea) according to the manufacturer’s protocol. The extracted DNA was used as the template for rRT-PCR. rRT-PCR was conducted using an Applied Biosystems™ 7500 Real-Time PCR device (Thermo Fisher^®^, Waltham, MA, USA) with the PowerChek™ Bovine Tuberculosis Real-Time PCR Kit (KogenBio^®^, Seoul, Republic of Korea). The PCR amplification conditions were as follows: an initial cycle at 50 °C for 2 min, followed by a denaturation step at 95 °C for 10 min, and 45 cycles of 95 °C for 15 s and 60 °C for 1 min.

#### 2.1.5. Statistical Analyses

SAS 9.4 (SAS Institute Inc^®^, Cary, NC, USA) was used to compare the concordance rates of the three diagnostic methods and to assess the differences between groups before and after PPD vaccination. The agreement between the three diagnostic methods was measured using the Kappa statistic. The bTB-positive cattle detected by TST and IFN-γ assays were divided into three groups, and a paired t-test was conducted to analyze differences in antibody value changes over time between the PPD inoculation day and seven days later.

### 2.2. Experimental Design

#### 2.2.1. Surveillance Period and Target Farms

From 2013 to 2017, three diagnostic tests (TST, IFN-γ, ELISA) were performed on 2355 cattle from 32 farms experiencing a bTB outbreak in Gangwon (Wonju, Hoengseong, Hongcheon). The detection rates and diagnostic agreement of the three tests were evaluated, and statistical analysis was performed to assess the correlation between diagnostic methods and the differences in ELISA results over time before and after PPD inoculation.

#### 2.2.2. Selection of the ELISA-Only-Positive Cattle in a bTB Outbreak Farm and PCR Confirmation

In 2013, three diagnostic methods—Tuberculin skin test (TST), IFN-γ assay, and ELISA—were performed on a farm in Ulju County, Gyeongsangnam-do, which housed 700 cattle (200 dairy cattle and 500 Hanwoo). This farm has reported a total of 200 cases of bovine tuberculosis over the past 10 years; however, it is not included among the 32 farms experiencing tuberculosis outbreaks in Gangwon-do as mentioned in Section 2.2.1 above. Lesion tissue samples (pharyngeal lymph node, pulmonary mediastinal lymph node) were collected from the ELISA-positive cattle for further rRT-PCR testing.

#### 2.2.3. Change of the ELISA Results Seven Days After PPD in TST and/or IFN-γ-Positive Cattle

The study included 111 cattle selected from 165 cattle that had previously tested positive for tuberculosis using TST and/or the IFN-γ assay. ELISA results were obtained for these 111 cattle both before and after PPD inoculation to assess ELISA s/*p* value changes. The ELISA test was performed on the day of PPD inoculation (first sample) and repeated seven days later (second sample). Blood samples were collected on both days to analyze changes in antibody levels, comparing the pre- and post-inoculation ELISA results.

#### 2.2.4. Changes in the ELISA Antibody Level Seven Days After PPD Inoculation in the Cattle Which Were All Negative for TST, IFN-γ Assay, and ELISA

Twenty cattle, all of which were negative for TST, IFN-γ, and ELISA, were selected from outbreak farms in the southern regions of Gangwon Province, South Korea. These cattle had been housed in a cohabitation space with bTB-positive cattle. ELISA S/*p* values were measured on the day of PPD inoculation (first sample) and again seven days later (second sample) to assess changes over time. To further investigate bTB infection, bTB lesion samples (lymph nodes) were collected from the cattle, and rRT-PCR was performed to confirm bTB infection in cases where the ELISA result converted to positive (S/*p* > 0.5) seven days after PPD inoculation.

#### 2.2.5. Application of ELISA for Eradication of bTB in the Chronic Outbreak Farms

To test the usefulness of ELISA when eradicating bTB in the outbreak farms, we chose 12 farms with chronic and recurrent outbreaks at 5 different areas of Gangwon province of the Republic of Korea. For this purpose, we classified 3 categories depending on the application protocol of the ELISA test for the detection of bTB. (A) Before November of 2013, ELISA was used for the first monitoring, followed by the second confirmation test, TST. (B) From December 2013 to November 2015, an ELISA test using the blood samples collected on the PPD inoculation day was performed together with TST and IFN-γ; for the duration, a cull recommendation was made for bTB-positive cattle with an S/*p* value of 0.5 or higher (S/*p* ≥ 0.5). (C) From December 2015 to June 2017, the ELISA test seven days after PPD inoculation was again performed to both TST and IFN-γ negative cases for culling; culling was undertaken in cases in which the ELISA value measured on the PPD inoculation day was higher than positive (S/*p* ≥ 0.5) and where the cattle were suspected of bTB infection and were housed in close contact with bTB-positive cattle.

## 3. Results

### 3.1. Detection Rate and Agreement of Three Diagnostic Methods in Thirty-Two bTB Outbreak Farms

As shown in Table 1, from 2013 to 2017, a total of 205 cattle (8.7%) out of 2355 cattle from 32 bTB outbreak farms were detected as bTB positive by at least one of the three diagnostic methods. The detection rate of ELISA was relatively low, at 52 cattle (2.2%), compared with TST (123 cattle, 5.2%) and IFN-γ (121 cattle, 5.1%). Additionally, the concordance of ELISA results with other methods was very low. ELISA results were concordant with only 12 cattle (0.51%) detected by IFN-γ and 8 cattle (0.34%) detected by TST. In contrast, the concordance rate between TST and IFN-γ was relatively high, at 79 cattle (3.69%). Furthermore, the number of positive cattle detected by all three methods was only eight (Table 1).

The three diagnostic methods (TST, IFN-γ assay, and ELISA) were tested concurrently on 2355 cattle from 32 bTB outbreak farms. The agreement between the three diagnostic methods was measured using the Kappa statistic.

The Kappa value for TST and IFN-γ assay was 0.628, indicating good agreement. In contrast, the Kappa value for TST and ELISA was 0.062, and for ELISA and IFN-γ assay, it was 0.111, both of which indicate poor concordance (Table 2).

### 3.2. The Twelve ELISA-Only-Positive Cattle in Chronic bTB Outbreak Farms

The three tests were performed three times, and a total of 17 bTB-positive cattle were ultimately detected. According to the results in Table 3, all cattle were tested negative by the TST; however, five cattle were found to be positive by the IFN-γ assay. Among the tested cattle, nine were strongly positive (S/*p* value > 0.5), and three were weakly positive (S/*p* value between 0.3 and 0.5), resulting in a total of twelve cattle that tested only positive by ELISA.

Of the 17 cattle that tested positive for tuberculosis, 12 cattle were positive only by ELISA, despite negative results in TST and/or IFN-γ assays. The ELISA S/*p* values of these 12 cattle ranged from 0.3 to 1.62, with a mean S/*p* value of 0.71.

An autopsy was performed on the 12 cattle, and pulmonary mediastinal lymph nodes were collected. The rRT-PCR test confirmed the final positive results.

Our findings demonstrate that 12 cattle, with an average S/*p* value of 0.3 or higher (doubtful or positive) in the ELISA test, were confirmed as true bTB-positive cases, even when TST and IFN-γ assay results were negative in these chronic bTB outbreak farms.

### 3.3. Change of the ELISA Results Seven Days After PPD in the TST and/or IFN-γ-Positive Cattle

**Figure 1:**
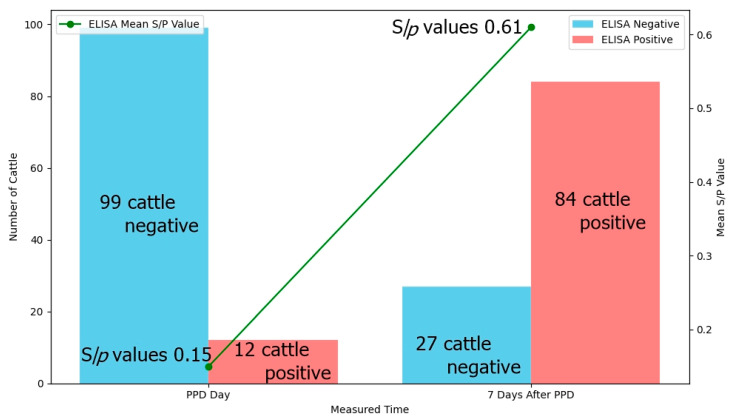
Change in the ELISA test results and mean s/*p* values obtained on the day of PPD inoculation and seven days after PPD inoculation in 111 cattle tested positive for TST and/or IFN-γ assay.

To investigate changes in ELISA results after PPD inoculation, ELISA was performed concurrently on 111 cattle among 165 cattle that tested positive by TST and/or IFN-γ from a total of 2355 cattle across 32 farms. The number of ELISA-positive cattle increased from 12 (11%) on the day of PPD inoculation to 84 (76%) on the seven days after PPD inoculation. Additionally, ELISA S/*p* values showed a significant increase from 0.15 to 0.61, shifting to a strong positive response. As shown in Figure 1, this seroconversion from ELISA-negative to ELISA-positive status in many cases led to a significant change in the agreement between ELISA and TST/IFN-γ results.

A total of 111 bTB-positive cattle, which were tested with ELISA before and after PPD inoculation, were classified into four groups based on the results: TST and IFN-γ both positive, IFN-γ only positive, TST only positive, and both TST and IFN-γ positive. Based on this, they were divided into the following four groups.: (A) Either TST or IFN-γ positive (111 cattle); (B) both TST and IFN-γ positive (62 cattle); (C) IFN-γ positive only (29 cattle); and (D) TST positive only (20 cattle).

A paired t-test using the SAS program was applied to statistically confirm the differences in ELISA S/*p* values between the day of PPD inoculation and seven days later in bTB-positive cattle.

**Figure 2:**
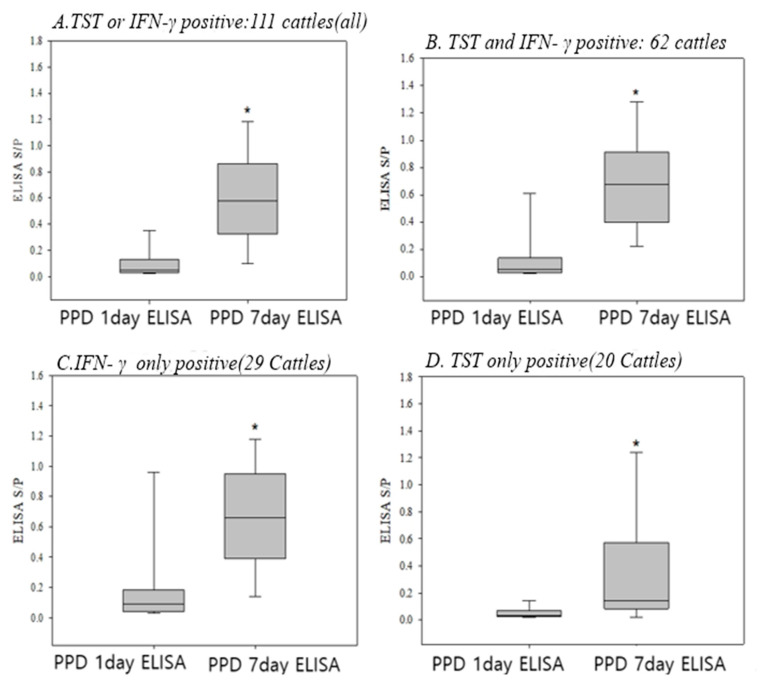
Boosting changes in blood antibody levels seven days after PPD inoculation in cattle showing positive for either TST, IFN-γ assay, or both (paired *t*-test). *, significantly different from the PPD 1day ELISA at *p* < 0.05.

The results show that ELISA S/*p* values significantly increased in all groups after PPD inoculation (Figure 2). In particular, the increase was more pronounced in cattle that were positive for both TST and IFN-γ and in those that were only IFN-γ positive (*p* < 0.0001), compared with cattle that were only TST positive (*p* < 0.05).

### 3.4. Conversion to Positive in the ELISA Results Seven Days After PPD in All Negative Cattle for TST, IFN-γ, and ELISA

A total of 20 cattle from 10 farms, all of which tested negative for TST, IFN-γ, and ELISA in bTB outbreak farms, were selected. These cattle converted to ELISA positive (S/*p* > 0.5) seven days after PPD inoculation.

The positivity of these cattle was further confirmed through rRT-PCR using lymph node tissue. Among the 20 ELISA-positive cattle, rRT-PCR tests were performed, and seven cattle from five farms were confirmed to be bTB positive.

Table 4 presents the ELISA results before and seven days after PPD inoculation, along with PCR confirmation in the seven ELISA-positive cattle. As shown, the ELISA results demonstrate a significant increase in S/*p* values, rising above 0.5 seven days after PPD inoculation.

### 3.5. Eradication of bTB in the Outbreak Farms Using the ELISA Test Seven Days After PPD Inoculation

As shown in Table 5, with this modified ELISA application, farms experiencing a bTB outbreak were more rapidly converted to bTB-negative status, as indicated by the notably reduced number of recurrences before eradication. Furthermore, recurrence was not observed to occur more quickly in outbreak farms that applied ELISA, TST, and IFN-γ simultaneously on the PPD inoculation day, compared with farms that used only TST and IFN-γ assays. Thus, ELISA performed on the PPD inoculation day together with TST and IFN-γ assays, followed by a second ELISA test seven days later, was confirmed to be the most effective detection method for bTB eradication. This approach enabled the detection and eradication of two to three times the average number of cases compared with other testing protocols.

## 4. Discussion

It has been a significant challenge to detect or eradiate bTB in South Korea, although three powerful methods, TST, IFN-γ, and ELISA have been applied for several decades [15,16]. The ELISA had been in use in South Korea from 2009 to 2013 and was excluded since 2013 because it had been assumed to generate false positives because of the low detection and agreement with TST results. As shown in Table 1 of this study, the detection rate of ELISA was just 2.2% on a sample base of 2355 heads of cattle in the 32 farms with bTB outbreaks. This was much lower than that seen with TST (5.2%) and IFN-γ assays (5.1%).

Some research papers published in South Korea during this period showed a low ELISA detection rate and low agreement with TST. Additionally, it is demonstrated in this study that the Kappa value of ELISA and TST or IFN-γ assays indicated <0.12, comparable to the much higher Kappa value of 0.650 between TST and IFN-γ assays (Table 2). The low detection rate of ELISA and agreement with the TST have generally been seen in cattle that underwent the single caudal fold skin test for the detection of bTB [20]. Because of this, ELISA was no longer in use and, instead, the IFN-γ assay had been adopted as another bTB detection method and has been used in combination with TST in Korea since 2013. Thereafter, TST and IFN-γ have been used simultaneously to detect bTB in the outbreak farms in order to undertake culls in South Korea [21]. However, despite vigorous efforts in culling bTB-positive cattle using TST and IFN-γ assay, some farms see a recurrence several months or even years later, making it difficult to convert them to bTB-negative farms [9].

Although there have been previous reports showing the low detection rate of bTB and the low concordance rate with TST, the usefulness of ELISA, established on the basis of humoral immune response, unlike the TST and IFN-γ assay, has not yet been clarified. Our present study demonstrates the usefulness of ELISA and provides a critical example of the eradication of bTB in the outbreak farms using the improved methodology of ELISA. First, our study showed that, although not always the case, ELISA-positive cattle (≥S/*p* value 0.5) and doubtful cattle (0.3 ≤ S/*p* value ≤ 0.5), which were negative for TST and IFN-γ, were confirmed to have been infected with bTB (Table 3), clearly indicating that the ELISA method could still be a useful method for the detection of bTB, in particular in the cases which were undetectable in the TST and IFN-γ. As suggested by previous reports, such a discrepancy of the results between TST and ELISA has been postulated to be due to the detection time point of bTB of the two methods; ELISA detects the late stage of bTB infection, characterized by increased antibody level, while TST detects the relatively early stage of bTB infection [1,2,3]. Our present study clearly showed that there must be cattle which are positive only for the ELISA test, but neither the TST nor the IFN-γ assay, in the bTB outbreak farms. This has also been reported in some previous studies, indicating that ELISA could detect bTB infected animals that had been missed by the TST and IFN-γ assays. In our study, the S/*p* values of those bTB-infected cattle which were positive only for ELISA, but not for either of the TST or the IFN-γ assays, ranged 0.3~1.62, taking an average of 0.63; here we need to pay attention to the significantly low S/*p* value, 0.3, in terms of potential cases with bTB infection (Table 3). Another important observation we made in the present study was that the ELISA S/*p* value can be significantly changed after PPD inoculation, something which is also strongly supported by previous studies [14,15].

According to our results, the ELISA S/*p* value was significantly increased seven days after PPD inoculation, not only in the 111 TST and/or IFN-γ-positive cases selected from the bTB outbreak farms, but also in the negative cases. The first ELISA value was measured on the day of PPD inoculation, and the second was evaluated seven days later, thus indicating the change in antibody level during the interim time period. Of these 111 heads of cattle, 72 cases (64%) showed a negative ELISA result when tested on the PPD inoculation day, but these cattle were converted to a positive result, showing a fourfold increase in the average ELISA antibody values seven days after PPD inoculation. Finally, the ELISA detection rates and agreements with other tests increased, thus negating the highlighted disadvantages of the ELISA test, as shown in Table 1. These changes were particularly dramatic in cattle showing positive for the IFN-γ assay, including Group A (either TST- or IFN-γ positive, *p* < 0.0001), Group B (both TST- and IFN-γ positive, *p* < 0.0001), and Group C (IFN-γ only, *p* < 0.0001), compared with those in the cattle positive for only Group D (TST only, *p* < 0.05) (Figure 2). The underlying mechanism explaining why those changes in the ELISA values occurred after PPD inoculation was not clarified in the present study; however, based on the results shown in Figure 2, our results provide clear evidence that ELISA measured seven days after PPD inoculation must be a very effective method for bTB detection in infected cattle which are undetectable by TST or IFN-γ assay or both [14,15,16,17]. In our study, the bTB infected cattle, which were undetectable for both TST and IFN-γ assays and represented negative or doubtful ELISA S/*p* values (≤0.3) when tested on the PPD inoculation day, dramatically increased to positive S/*p* values (≥0.5) seven days after the PPD inoculation, as shown in Table 4. Notably, these seven cattle were housed together with truly negative cattle. These cattle, which were not detected by the three diagnostic methods in bTB outbreak farms, could act as vectors for transmission. It was an unexpected surprise, discovered in the present study, that bTB infected cattle showing such low S/*p* values of 0.01~0.03, which were also negative for TST and IFN-Γ γ assay, were detectable by ELISA when measuring on day seven after the PPD inoculation, as the S/*p* values were highly increased and had changed into positive values (≥0.5). Such ELISA-boosting-positive cases (cattle that initially test negative but turn ELISA positive after seven days) have frequently been observed in farms where a high number of cattle test positive for TST and IFN-γ (≥30%), or TB has been endemic for an extended period (chronic TB-positive farms). The case described in Table 4 represents one such instance, where an animal that was initially undetectable by standard cell-mediated immune tests later exhibited a boosted ELISA response. Those results may explain why there has been a failure to eradicate bTB in Korea, especially in the outbreak farms where bTB occurred more than three times or where more than one third of the housed cattle have been infected with bTB (Korean QIA reference). In addition, the results strongly support the usefulness of ELISA for the eradication of bTB in such frustrating farms.

The increasing changes of ELISA S/*p* values after PPD inoculation has also been shown in the previous study [16], in which the responses of antibodies specific to various antigens, especially MPB83, significantly increased two weeks after the skin test in the experimentally unvaccinated bTB infected cattle, depending on the disease severity and bacterial load. The boosting effect of TST in the bTB-infected cattle was demonstrated again in this study, in the 111 cases positive for TST and/or INF in the bTB outbreak farms [14,15,16,17]. In addition, it was suggested that blood collection should be undertaken 7 to 60 days after caudal-fold tuberculin (TST) inoculation, specifically between 7 and 14 days to quickly diagnose latent bTB infection. This method improved the sensitivity and accuracy of the specific ELISA (IDEXX M. bovis Ab test) as a method for bTB diagnosis. In other papers published globally, it was confirmed that the ELISA test performed 7 to 14 days after the PPD inoculation was better at detecting bTB-positive cattle. The best timing of the ELISA test after PPD inoculation seems to be dependent on the ELISA kit used and various factors such as the animal’s immune status. In our study, 7~10 days post-tuberculin test was found to be efficient for the second ELISA test, when using the bTB 2.0^®^, BIONOTE [15].

Taken together, our results could be summarized as follows: Our findings suggest that bTB-positive cattle undetectable by TST and IFN-γ assays can be identified via ELISA seven days after PPD inoculation. In these cases, bTB infection was confirmed via rRT-PCR analysis of lesion samples. Finally, we propose a new ELISA-based protocol that facilitates bTB eradication with minimal culling and demonstrate its effectiveness in controlling outbreaks.

First, the cattle could be bTB-infected cases, showing ELISA S/*p* values of more than 0.5, or highly suspicious, showing an S/*p* value ≥ 0.3, when measured on the day of TST, even where they are all negative for both TST and IFN-γ assay; second, for the cattle showing low negative S/*p* values (≤0.5) as well as all negative values for TST and IFN-γ, if ELISA S/*p* values are highly increased and change to positive values (≥0.5) seven days after the PPD inoculation, they could be bTB-infected cattle and able to play a role in spreading bTB in the farms without culling. We propose a new ELISA-based protocol that facilitates bTB eradication with minimal culling and demonstrate its effectiveness in controlling outbreaks. Based on our results, we attempted to develop criteria for the use of ELISA to detect bTB, in particular in the bTB-infected cattle, which are undetectable by TST and/or IFN-γ assay, and tried to apply the criteria to eradicate bTB in the outbreak farms (Table 5).

**Figure 3:**
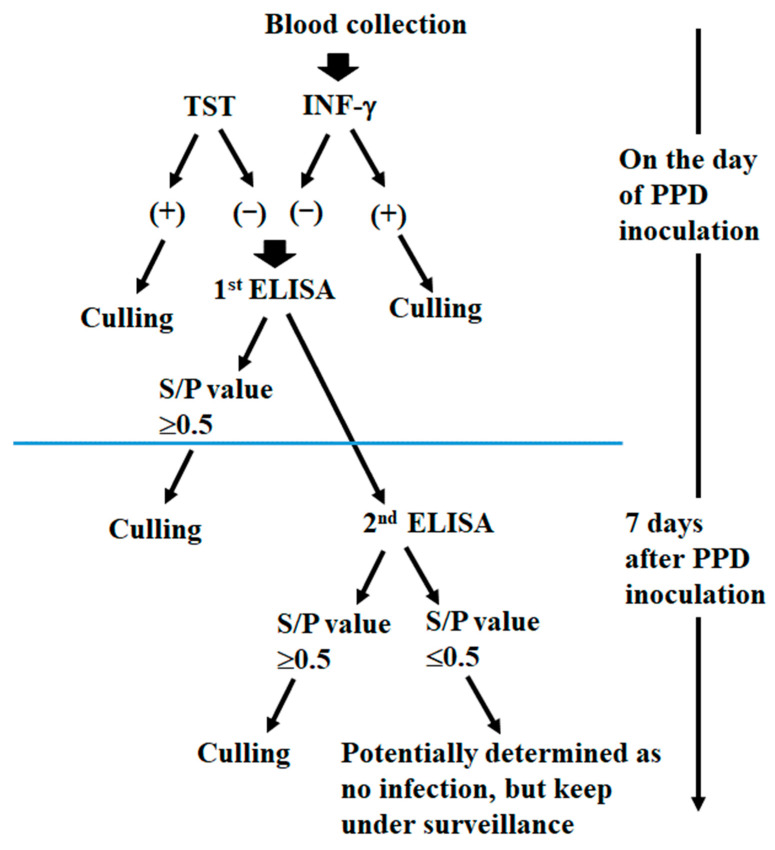
Bovine tuberculosis eradication strategy using ELISA testing.

We classified three subsequent steps for the application of ELISA for the detection of bTB (Figure 3); as a first step, cattle showing positive for either the TST or the IFN-Γ γ assay are sought on the PPD inoculation day. As a second step, ELISA is performed on the day of PPD inoculation, and the cattle showing an S/*p* value of 0.5 or higher and the TST- and/or IFN-Γ-γ-positive cattle suspected to be infected with bTB are set aside for culling. Cattle with an S/*p* value of ≥0.3 and ≤0.5 in the ELISA are excluded from culling at this stage. However, since these cattle have been housed in close proximity to bTB-positive cattle, they could potentially play a role as latent infected individuals (Table 3). In this step, ELISA could be optionally performed only in the cattle which are negative for TST and IFN-Γ γ assay. As the third step, ELISA will be performed again, seven days after PPD inoculation, for the cattle which are all negative for TST, IFN-Γ γ, and ELISA. A cull recommendation is then made for the bTB-positive cattle with an S/*p* value ≥ 0.5 in the second ELISA application (Table 4).

In the present study, we applied this critical application protocol of ELISA to the 12 bTB outbreak farms in Gangwon province of South Korea, in order to investigate how to efficiently eradicate bTB. The farms chosen belonged to five different areas, having chronic and recurrent bTB outbreaks. Epidemiological investigations revealed that there were several risk factors for the spread of bTB in those farms, such as purchase of cattle from outside, transmission from farms with tuberculosis outbreaks to neighboring farms, and so on. The HS-2 area was the largest bTB outbreak area in Gangwon province, with 200 bTB-positive heads of cattle. The HS-2 area had several additional risk factors, including common water sources, deer breeding, and the use of shared equipment. The risk factors associated with bovine tuberculosis (bTB) outbreaks in this study were identified through an epidemiological survey, with external purchase of cattle and transmission from neighboring farms being the main causes [22,23]. The farms targeted in this study for bovine tuberculosis consisted of a total of 33 farms, with 29 farms (88%) being Korean cattle (Hanwoo) farms and 4 farms (12%) being dairy farms. As the majority of the farms were Hanwoo farms, further investigation into the dairy farms is necessary due to genetic differences in susceptibility to bTB [24,25,26,27].

As shown in Table 5, the simultaneous application of ELISA with the other two tests helped to convert bTB farms to bTB-negative farms. The farms that simultaneously apply ELISA, TST, and IFN-γ assay on the PPD inoculation day could eradicate bTB faster than the farms that used only two methods, TST and IFN-γ assay. In the farms that used ELISA with TST and IFN-γ assays simultaneously on PPD inoculation day and again seven days after PPD inoculation, bovine tuberculosis recurrence dropped by an average of two to three times. Thus, the eradication of bTB was much faster in the outbreak farms when ELISA was simultaneously applied with TST and IFN-Γ γ assays, compared with the farms that used only the TST and IFN-γ assays. In conclusion, our present study clearly demonstrated, in the field, that ELISA is a pivotal tool for the fast and efficient eradication of bovine tuberculosis. This is achieved by the application of the newly developed protocol, including the measurement of the secondary ELISA on seven days after PPD inoculation.

## Figures and Tables

**Table 1:** Detection rate and concordance of each diagnostic method in bTB outbreak farms (*n* = 2355 cattle from 32 farms).

Group	Positivity for Bovine Tuberculosis			No. of Cattle (%)	No. of Positive Cattle for Each Method (Detection Rate, %)		
	TST	IFN-γ	ELISA		TST	IFN-γ	ELISA
Negative	−	−	−	2150 (91.3)	0	0	0
TST only	+	−	−	44 (1.9)	44	0	0
IFN-γ only	−	+	−	38 (1.6)	0	38	0
ELISA only	−	−	+	40 (1.7)	0	0	40
TST and IFN-γ	+	+	−	79 (3.4)	79	79	0
TST and ELISA	+	−	+	8 (0.3)	8	0	8
IFN-γ and ELISA	−	+	+	12 (0.5)	-	12	12
TST and IFN-γ and ELISA	+	+	+	8 (0.3)	8	8	8
Total positivity 205 (8.7) *				2355 (100) *	123 (5.2) *	121 (5.1) *	52 (2.2) *

TST, Tuberculin skin test; IFN-γ, interferon-gamma; ELISA, enzyme-linked immunosorbent assay. *, duplicate cattle were regarded as one case.

**Table 2:** Agreement between the three diagnostic methods in bTB outbreak farms.

		**TST**				**ELISA**				**ELISA**	
		−	+			−	+			−	+
**IFN-γ**	−	2190	44	**TST**	−	2188	44	**IFN-γ**	−	2194	40
	+	42	79		+	115	8		+	109	12
Overall agreement 96.3%				Overall agreement 93.2%				Overall agreement 93.7%			
Kappa value 0.628				Kappa value 0.062				Kappa value = 0.111			

*TST, Tuberculin skin test; IFN-γ, interferon-gamma; ELISA, enzyme-linked immunosorbent assay.*

**Table 3:** Confirmation of bovine tuberculosis infection in 12 cattle that tested positive only by ELISA, but negative by TST and/or IFN-γ assay, in chronic BTB outbreak farms.

Animal ID	TST	IFN-γ	ELISA *	Range of the ELISA S/*p* Values	ELISA Mean S/*p* Value	rRT-PCR
H2	−	−	+	0.68~1.62	1.17	+
H12	−	−	+	0.81~1.12	1.04	+
H5	−	−	+	0.53~1.27	0.95	+
H39	−	−	+	0.67~1.12	0.91	+
H20	−	−	+	0.74~0.98	0.88	+
H14	−	−	+	0.33~0.81	0.61	+
H1	−	−	+	0.49~0.70	0.60	+
H9	−	−	+	0.32~0.68	0.55	+
H38	−	−	+	0.35~0.71	0.54	+
H17	−	−	±	0.36~0.67	0.42	+
H22	−	−	±	0.31~0.66	0.41	+
H54	−	−	±	0.3~0.74	0.39	+
**Total**	−	−	**12**	**0.3~1.62**	**0.71**	**12 positive**

rRT-PCR was performed using the pulmonary lymph node of each animal *; −, negative (S/p value ≤ 0.3); ±, doubtful (0.3 ≤ S/p value ≤ 0.5); +, positive (S/p value ≥ 0.5) TST: Tuberculin skin test; IFN-γ: interferon-gamma; ELISA: enzyme-linked immunosorbent assay.

**Table 4:** ELISA results before and seven days after PPD inoculation in seven cattle that tested TST and IFN-γ negative.

Farm ID	Positive Cattle Age	TST Result	IFN-γ Result	ELISA S/*p* Value (PPD Inoculation Day)	ELISA S/*p* Value (Seven Days After PPD)	rRT-PCR
J○○	89 months	−	−	0.47	1.11 (+)	+
L○○	48 months	−	−	0.36	1.21 (+)	+
L○○	15 months	−	−	0.49	0.77 (+)	+
Y○○	35 months	−	−	0.29	0.51 (+)	+
U○○	69 months	−	−	0.02	1.33 (+)	+
U○○	65 months	−	−	0.01	0.67 (+)	+
K○○	54 months	−	−	0.03	0.97 (+)	+

*TST, Tuberculin Skin Test; IFN-γ, Interferon-gamma; ELISA, Enzyme-linked Immunosorbent Assay.*

**Table 5:** Comparison of bTB recurrence in farms using the modified ELISA protocol versus farms using traditional methods.

Study Period	Area	Farm ID	Breed	Herd Size	No. of bTB-Cattle	Application Protocol	No. of bTB Recurrences
until 2013.11	HS-1	U○○	Hanwoo	71	38	TST only confirm (ELISA monitoring)	8
		P○○		69	18		5
2013.12~2015.11	HC	J○○		74	3		3
	HC	J○		16	7	Simultaneous 3 Test TST, IFN-γ and ELISA (seven days post-PPD)	4
		J○○		34	22		4
	HS-2	S○○	Dairy cow	42	8		4
	**SUM**			**306**	**96**	**MEAN**	**4.7**
2015.12~2017.06	HC	J○○	Hanwoo	124	8	Simultaneous 3 Test TST, IFN-γ and ELISA (seven days post-PPD)	2
	HS-2	B○○		75	6		1
		Y○○		66	4		1
		U○○		157	19		2
	HS-3	L○○		142	43		2
	HS-4	K○○		47	34		2
	**SUM**			**611**	**114**	**MEAN**	**1.7**

## Data Availability

The data presented in this study are contained within the article.
